# Infiltration of thyroid papillary cancer tissue with myeloid leukemic cells: a case report

**DOI:** 10.1186/s12957-021-02341-z

**Published:** 2021-07-29

**Authors:** Mehmet Sözen, Çiğdem Vural, Alev Selek, Umay Kiraz, Zeynep Cantürk, Berrin Çetinarslan, Emre Gezer, Damla Köksalan

**Affiliations:** 1 Department of Endocrinology and Metabolism, Kocaeli University Faculty of Medicine, İzmit, Kocaeli Turkey; 2 Department of Pathology, Kocaeli University Faculty of Medicine, İzmit, Kocaeli Turkey

**Keywords:** Myeloid sarcoma, Breast, Thyroid, Papillary thyroid carcinoma

## Abstract

**Background:**

Extramedullary leukemia, also known as myeloid sarcoma, is a rare form of acute myeloid leukemia and often accompanies bone marrow involvement. Myeloid infiltration of the thyroid gland is extremely rare. Here, a unique case in which thyroid cancer tissue was infiltrated with myeloid cells is presented.

**Case presentation:**

We present a case of thyroid papillary cancer infiltrated by blastic cells and bilateral breast and axillary myeloid sarcoma in a 30-year-old Caucasian female patient with a history of osteosarcoma and MDS-RAEB2. The patient firstly received 6 cycles of chemotherapy for osteosarcoma, and allogeneic hematopoietic stem cell transplantation was performed after anthracycline-based chemotherapy due to MDS-RAEB2. The patient remained in remission on follow-up in terms of both osteosarcoma and MDS-RAEB2. Malignant features (Bethesda VI) were observed in the fine needle aspiration biopsy performed from a newly developed firm, fixed thyroid nodule approximately 4–5 cm in length in the left thyroid lobe. Because of the Bethesda VI thyroid nodule, the patient underwent total thyroidectomy. In the pathological evaluation, CD34-, CD117-, MPO-, and HLA-DR-positive blastic cells which infiltrated into follicular variant papillary thyroid carcinoma were detected. In the evaluation performed due to blastic cell infiltration, multiple lesions showing increased 18-fluorodeoxyglucose activity in bilateral breast and axillae were detected. Myeloid sarcoma was found as a result of tru-cut biopsy from these lesions. A fungal cystic lesion was detected in the frontal region of the patient who developed altered consciousness after the second cycle of treatment of myeloid sarcoma. During her follow-up in the intensive care unit, she died of cranial septic embolism and acute infarction.

**Conclusions:**

Here, we present a very interesting case that is the first. A staged approach to diagnosis with methods including immunohistochemical staining, radiological imaging methods, and cytogenetic and molecular analyses can help make the definitive diagnosis.

## Background

The papillary thyroid carcinoma (PTC) is characterized pathologically by papillary architecture and typical nuclear features of chromatin pallor, nuclear enlargement, grooves, and pseudoinclusions. Follicular variant PTC (FVPTC) is the second most common subtype of the PTC family, accounting for 9–20% of patients. This variant has a follicular growth pattern along with the typical nuclear properties of PTC. Environmental, genetic, and hormonal factors play a role in the etiology of PTC [1]. PTC has a relatively good prognosis, although an anaplastic transformation of a well-differentiated PTC may rarely occur and is associated with poor prognosis [2].

Myeloid sarcoma (MS) was first described by Burns in 1811. Later, in 1966, Rappaport proposed the term “granulocytic sarcoma” for this tumor. The World Health Organization declared the name “myeloid sarcoma” in 2002 [3]. MS may develop as part of acute myeloid leukemia, myeloproliferative diseases, or myelodysplastic syndrome (MDS) and may occur especially during recurrence following allogeneic hematopoietic stem cell transplantation (HSCT) [4, 5]. MS is commonly seen in the soft tissues, bones, central nervous system, and lymph nodes. However, it is rarely located in organs such as the eyes, gonads, and breasts [6].

Here, we report a case of isolated MS occurring in the breast and thyroid without bone marrow involvement after a successful allogeneic hematopoietic stem cell transplantation. The interesting aspect of this case is that while leukemic involvement in the breast is in favor of a complete myeloid sarcoma, thyroid involvement is only characterized by leukemic infiltration. What makes the case even more interesting is that the leukemic infiltration in the thyroid gland was seen only within the follicular variant papillary thyroid carcinoma that contained concurrent focal anaplastic differentiation areas.

## Case presentation

A 30-year-old Caucasian female patient presented with the complaint of a newly developed mass in the neck. The patient’s marital status was single and parity was 0. The patient’s family history was unremarkable. Her past medical history revealed that she was operated due to osteosarcoma of the left tibia distal end in 2003 and given 6 cycles of chemotherapy (ifosfamide + mesna + adriamycin). Borderline phyllodes tumor was detected in tru-cut biopsy performed due to the detection of a mass in the right breast in 2016 and it was totally excised. At the same period, pancytopenia was seen in complete blood count, and on further evaluation, target cells, acanthocytosis, and thrombocytosis were detected in the peripheral blood smear. In the bone marrow aspiration smear and biopsy, megaloblastic changes, severe erythroid dysplasia, and blastic infiltration (18%) were detected. In the genetic examination performed with real-time polymerase chain reaction, t(8,21) and inv(16) were found to be negative. Cytogenetic analysis performed by fluorescent in situ hybridization revealed 21% amplification of mixed lineage leukemia (MLL) gene at chromosome 11q23 and 10% trisomy 17. The patient was diagnosed with myelodysplastic syndrome-refractory anemia with excess blasts 2 (MDS-RAEB2) and anthracycline-based induction chemotherapy was given, and allogeneic HSCT was then carried out. The patient remained in remission on follow-up in terms of both osteosarcoma and MDS-RAEB2; however, there was no follow-up for possible recurrence of the tumor in the breast.

**Fig. 1:**
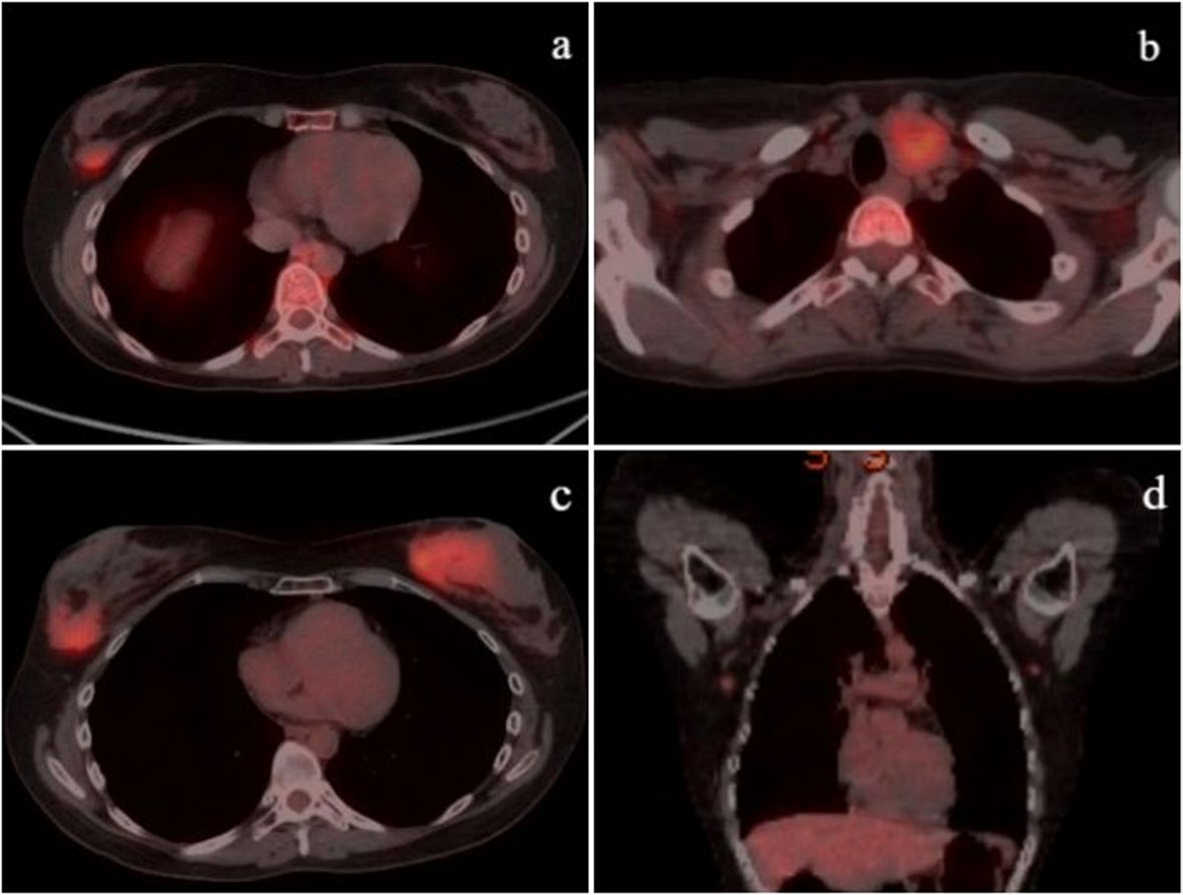
FDG-PET images of breast and thyroid lesions. **a** Lesion showing increased FDG uptake in the right mammary gland at diagnosis (size 2 cm, SUV max 3.9). **b** Lesion showing increased FDG uptake (SUV max 5.7) in the left lobe of the thyroid gland at diagnosis. **c** Lesion in the right breast showing increased size and metabolism compared to previous FDG-PET (size 4.2 cm, SUV max 9.6) and a newly developed lesion in the left breast. **d** Lymphadenopathies showing increased FDG uptake in both axillae (size 1 cm, SUV max 3.7)

After admission to our clinic, a firm, fixed thyroid nodule approximately 4–5 cm in length extending to the supraclavicular area was detected on the left thyroid lobe. Bilateral breast examination revealed an approximately 1–2-cm moveable lesion in the left breast at 7 o’clock position and a 3-cm moveable lesion in the right breast at 8 o’clock position. Other physical examination findings were normal. In the laboratory evaluation, the patient was euthyroid. Thyroid ultrasonography (USG) revealed a 26.5 × 24 × 43 mm nodule containing cystic areas with a thin hypoechoic halo in the left lobe. Malignant features (Bethesda VI) were observed in fine needle aspiration biopsy performed from that nodule. On the breast USG, a heterogeneous mass with a size of 25 × 14 mm with uncertain borders was observed in the right lower outer quadrant. In addition, few bilateral subcentimetric hypoechoic lesions were observed. 18-Fluorodeoxyglucose-positron emission tomography (FDG-PET) imaging was performed due to the history of multiple malignancies. In FDG-PET imaging, a 2 × 1.5 cm lesion showing increased FDG uptake (SUV max 3.9) in the lower outer quadrant of the right breast (Fig. 1a) and a lesion showing increased FDG uptake (SUV max 5.7) in the left thyroid lobe were observed (Fig. 1b).

**Fig. 2:**
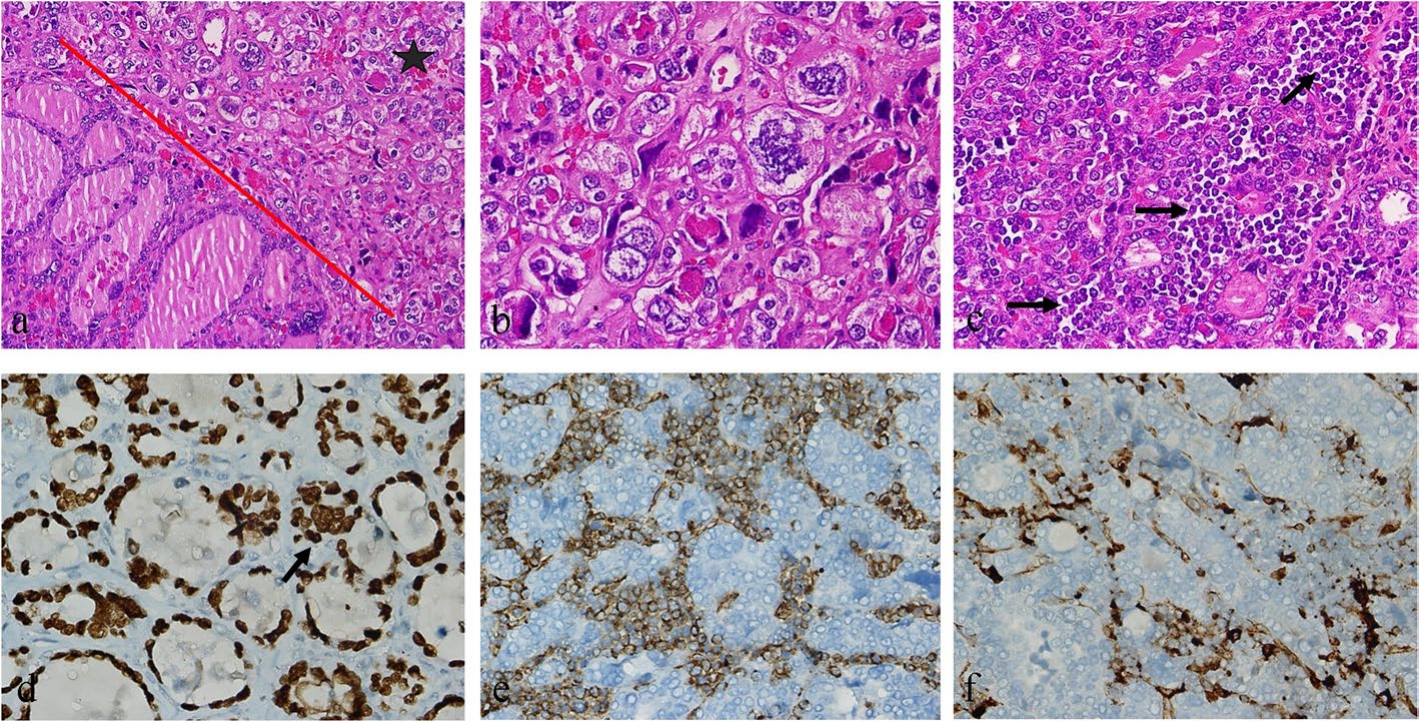
Blastic cell infiltration in FVPTC. **a** FVPTC with focal anaplastic differentiation areas (H.E. × 200). The star side of the red line consists of anaplastic cells, and the other side is the FVPTC part. **b** Anaplastic cells (H.E. × 400). **c** Blastic cell infiltration between follicles (H.E. × 400). Arrow: blastic cell groups. **d** Immunohistochemical staining of follicular cells and anaplastic cells showing diffuse strongly nuclear positive for TTF-1 (× 400). Arrow: anaplastic cell. **e** Immunohistochemical staining of blast cells showing diffuse, strongly cytoplasmic positive for CD34 (× 400). **f** Immunohistochemical staining of blast cells showing focally strongly cytoplasmic positive for HLA-DR (× 400)

**Fig. 3:**
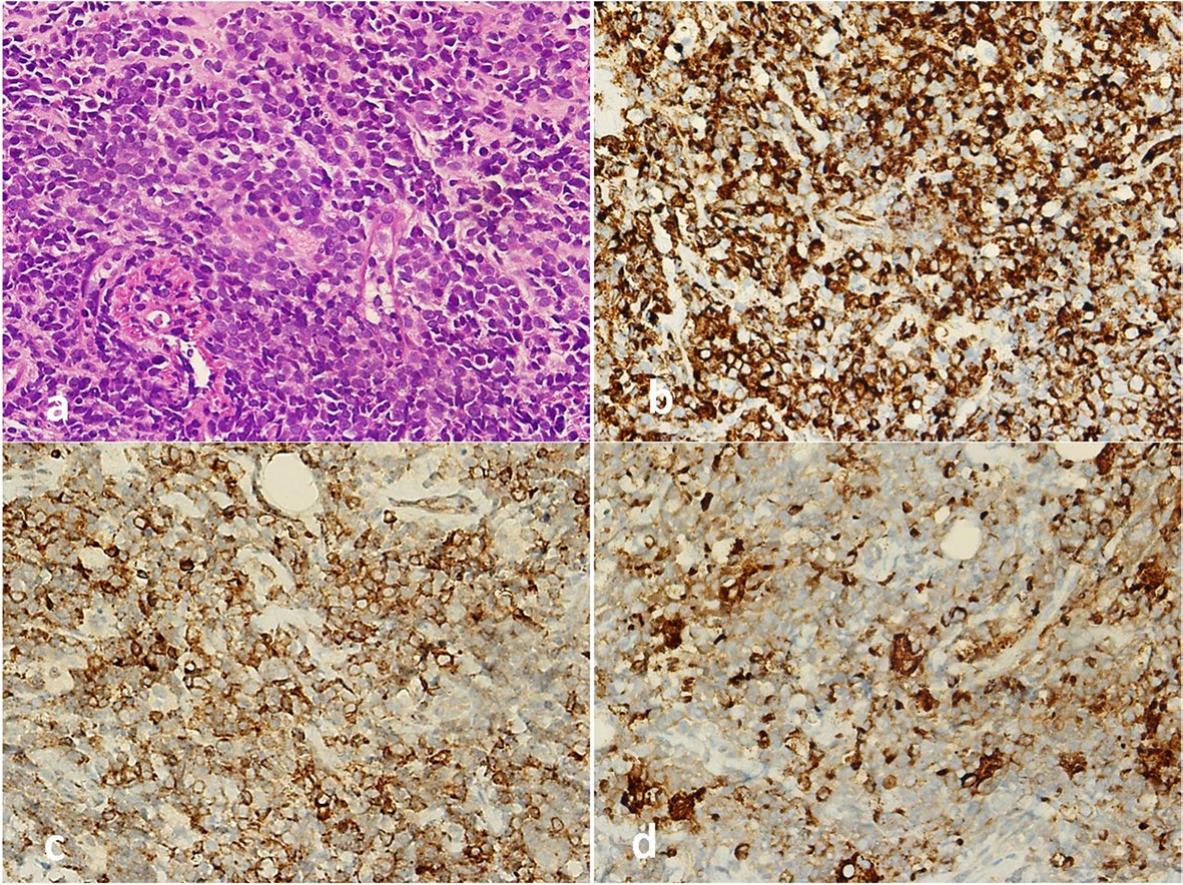
Blastic cell infiltration in lesions of the breast and axilla. **a** Diffuse blastic cell infiltration in breast tissue (H.E. × 400). **b** Immunohistochemical staining of blast cells showing diffuse, strongly cytoplasmic positive for CD34 (× 400). **c** Immunohistochemical staining of blast cells showing diffuse, strongly cytoplasmic positive for CD117 (× 400). **d** Immunohistochemical staining of blast cells showing focally strongly cytoplasmic positive for MPO (× 400)

The patient underwent total thyroidectomy, and pathological macroscopic examination revealed a solid, off-white colored nodule with a size of 5 × 3.5 cm completely filling the left lobe. In hematoxylin–eosin staining, the nodule had an encapsulated appearance, infiltrated as nodular tumor masses outside the capsule, and it was consisted of follicular epithelial cells with prominent PTC nuclear features, which formed mostly follicular structures. In approximately 15–20% of the nodule, anaplastic cells were seen, which were generally distributed one by one among the thyrocytes, and separated from the PTC areas with a sharp border in places. Since these anaplastic cells did not constitute the dominant tumor component in the nodule, they were evaluated in favor of focal areas of dedifferentiation. In addition, blastic cells that did not form a mass in the nodule were also observed. Immunohistochemically, diffuse staining with CD34, as well as less frequent staining with myeloperoxidase (MPO) and HLA-DR, was observed in these blastic cells. Considering all these findings, the nodule in the left lobe was defined as a stage II (T3aN0M0) follicular variant of PTC (FVPTC) with focal anaplastic differentiation areas and blastic cell infiltration (Fig. 2). There were no blastic cells in the peripheral blood smear and bone marrow aspiration smear. FDG-PET revealed that the size and metabolism of the previously existing right breast mass were increased (Fig. 1c). In addition, new FDG-positive lesions were detected in the left breast (Fig. 1c) and both axillae (Fig. 1d). The tru-cut biopsy performed from bilateral breast and axillary lesions showed diffuse blastic cell infiltration. Immunohistochemically, diffuse staining with CD34, CD117, MPO, and HLA-DR was observed in these blastic cells. These results were evaluated in favor of myeloid sarcoma (Fig. 3). p53 gene analysis that was performed for possible Li-Fraumeni syndrome due to soft tissue sarcoma and MDS-RAEB2 was negative.

The patient was evaluated in the multidisciplinary council, and 150 mci radioactive iodine (RAI) treatment was given for thyroid papillary cancer. Stimulated thyroglobulin during RAI treatment was found to be 1.76 ng/mL. 1.6 mcg/kg/day levothyroxine was started 2 days after RAI treatment. After this treatment, FLAG-IDA (fludarabine, cytarabine, idarubicin, G-CSF) chemotherapy treatment was planned in the hematology department for the treatment of myeloid sarcoma. Cranial imaging performed upon the development of confusion and somnolence after two cycles of FLAG-IDA treatment revealed a lesion consistent with an abscess in the right frontal region. The culture of the abscess drainage material was positive for fungal infection, and intravenous amphotericin B treatment was therefore initiated during the follow-up in the intensive care unit (ICU). In the follow-up of the patient in ICU, who responded to the current treatment and suddenly developed bilateral vision loss and brain MRI showed multiple septic embolism foci and acute infarct foci in the right cerebral hemisphere and occlusion in the right internal carotid artery, the patient died in ICU due to multiple organ failure.

## Discussion

Metastasis of solid organ tumors to the breast and thyroid gland is extremely rare. Metastatic tumors of the breast account for only 0.3–2% of all breast lesions and metastasis to the thyroid is seen in 2–3% of thyroid malignant lesions [7, 8]. Isolated case reports of breast and thyroid involvement in acute and chronic leukemias have emerged [9, 10]. In this article, a patient with blast cell infiltration in thyroid papillary carcinoma tissue and breast myeloid sarcoma without bone marrow involvement was presented.

High-risk MDS patients may develop MS representing the leukemic transformation, and this leukemic transformation occurs in approximately 15 to 17% of patients at 5 to 6 years [11]. MS seems to develop more frequently in patients with RAEB. The risk of conversion to leukemia in these patients was 25 and 33% for RAEB1 and RAEB2, respectively [12]. The incidence of recurrence of MS after allogeneic HSCT ranges from 0.65 to 7.4% [13]. After HSCT, bone marrow recurrence occurs within 3–6 months, while extramedullary relapse occurs within 12–17 months [13–15]. Rarely, extramedullary relapses can be seen 5–10 years after HSCT [14]. In our case diagnosed with RAEB2, isolated extramedullary recurrence occurred 46 months after the diagnosis of MDS and allogenic HSCT. While this was a predictable time for leukemic transformation of MDS, it was a fairly long time for relapse after HSCT.

Thyroid myeloid sarcoma is a rare condition. In a Japanese series of 131 MS patients, only one patient had MS located in the thyroid [16]. Blastic cell infiltration in the thyroid gland is extremely rare, and diffuse blastic infiltration of the thyroid is accompanied by bone marrow involvement in these cases [17, 18]. The interesting finding in this case was that the blastic cells in the thyroid gland were infiltrating only a nodule that was consisted of entirely thyroid cancer cells. There were no blastic cells on the remaining thyroid gland. The diffuse staining of these blastic cells with CD34, MPO, and HLA-DR suggested their myeloid origin. Intratumoral anaplastic transformation from pre-existing differentiated thyroid carcinomas has become a well-known process, despite limited knowledge of underlying mechanisms [2]. Cells in approximately 15–20% of the nodule were stained positively with TTF1 and thyroglobulin. Vimentin was negative when examined for a possible sarcomatoid metastasis due to a history of osteosarcoma. This suggested the thyroid origin of the cells. These cells were evaluated in favor of anaplastic differentiation due to their morphological multinuclear bizarre appearance. To the best of our knowledge, it is the first case of myeloid blastic cell infiltration into thyroid cancer tissue.

MS pathophysiology is still not clearly understood, but altered homing mechanisms for myeloid serial cells are to blame. Abnormal expression of chemokine receptor subtypes by MS blasts, leukemic cell surface markers such as CD56, and protease-mediated interaction with endothelial cells may be responsible for the increased invasiveness of leukemic clones [19, 20]. In cytogenetic examinations performed in MS patients, various cytogenetic abnormalities have been detected in more than half of the cases. MS is well-described in patients with recurrent genetic abnormalities, including t(8;21), inv(16), t(16;16), MLL rearrangements, trisomy 8, monosomy 7, FLT3-ITD, and NPM-1 mutation [21, 22]. MLL gene amplification and trisomy 17 detected in our patient support the role of cytogenetic abnormalities in the pathophysiology of MS.

Mutations involving effectors of the mitogen-activated protein kinase (MAPK) pathway such as RET, BRAF, RAS, and NTRK1 occur in approximately 70% of patients with PTC and are associated with histopathological and biological tumor behavior [23]. In the genomic analysis of PTC, many low frequency genetic changes have been identified, the importance of which is not fully understood. Determining which genes show more mutations than expected and which of the genes is a “driver” rather than a “passenger” is an important step in carcinogenesis. These low frequency genes are of great interest as they encode proteins involved in pathways and functional groups involved in carcinogenesis. In the PTC genomic analysis, 20% of tumors showed rare mutations within epigenetic regulatory genes, including mutations in MLL (1.7%), ARID1B (1.0%), and MLL3 (1%) most commonly [24]. In this case, both the development of thyroid cancer and the development of leukemic cell infiltration in thyroid cancer tissue may be associated with the MLL gene mutation.

In a series of 96 MS cases reported by the Mayo Clinic, only 3% of cases with breast localization were detected [25]. MS of the breast can occur as a unilateral or bilateral mass without nipple retraction. MS with bilateral breast involvement was detected only in a few cases in the literature [9, 26]. It is known that MS can occur with lymph node involvement, although it is uncommon [27]. Diagnosis of MS is challenging due to its histological and radiological similarities with malignant lymphoma and its variable morphology. In such cases, appropriate immunohistochemical studies are required to reach the correct diagnosis. Myeloid sequence markers such as MPO, CD34, and CD117 should be used for diagnosis [28]. Our case was thought to be in favor of myeloid sarcoma, since MPO-, CD34-, CD117-, HLA-DR-, and LCA-positive immunoreactivity was detected in the tru-cut biopsy specimens performed from lesions showing FDG uptake in bilateral breasts and axillae. To the best of our knowledge, we present the first case of bilateral axillary involvement concurrent with the breast.

Centers of excellence that focus on specific medical areas provide comprehensive and multidisciplinary in-depth expertise, and relevant resources provide many benefits for healthcare providers and the populations they serve. These centers are also capable of delivering better results through the application of innovative methods and techniques that improve outcomes [29]. Regular multidisciplinary clinical follow-up is very important for early diagnosis in such a patient with a high cancer burden and genetic mutation rate. However, keeping in mind the complications that may develop at any time and patient follow-up with a multidisciplinary team have an important place in patient management.

## Conclusion

MS can present as a leukemic transformation of MDS. Isolated MS without bone marrow involvement is extremely rare and histopathological examination is a key factor in diagnosis. In this case, we describe a case of thyroid papillary cancer tissue infiltrated by leukemic cells. This situation is quite interesting and shows the feature of being the first in the literature. In addition, the patient has simultaneous bilateral breast and axillary MS. A staged diagnostic approach with diagnostic tools including an extensive differential diagnosis, clinical suspicion, immunohistochemical staining, and radiological imaging methods such as FDG-PET, as well as cytogenetic and molecular analyses, may help to facilitate the establishment of the final diagnosis.

## Data Availability

The datasets used are available from the corresponding author on reasonable request.

## Abbreviations

PTC: Papillary thyroid carcinoma
FVPTC: Follicular variant papillary thyroid carcinoma
MS: Myeloid sarcoma
MDS: Myelodysplastic syndrome
HSCT: Hematopoietic stem cell transplantation
MDS-RAEB2: Myelodysplastic syndrome-refractory anemia with excess blasts 2
USG: Ultrasonography
FDG-PET: 18-Fluorodeoxyglucose-positron emission tomography
MPO: Myeloperoxidase
FLAG-IDA: Fludarabine, cytarabine, idarubicin, G-CSF
ICU: Intensive care unit
